# Early evolution of polyisoprenol biosynthesis and the origin of cell walls

**DOI:** 10.7717/peerj.2626

**Published:** 2016-10-26

**Authors:** Jonathan Lombard

**Affiliations:** 1 Biosciences, University of Exeter, Exeter, United Kingdom; 2 National Evolutionary Synthesis Center, Durham, NC, United States of America

**Keywords:** Polyisoprenol, Lipid carrier, Cenancestor, LUCA, Cell walls, Glycosylation, Archaea, Bacteria, Eukaryotes

## Abstract

After being a matter of hot debate for years, the presence of lipid membranes in the last common ancestor of extant organisms (i.e., the cenancestor) now begins to be generally accepted. By contrast, cenancestral cell walls have attracted less attention, probably owing to the large diversity of cell walls that exist in the three domains of life. Many prokaryotic cell walls, however, are synthesized using glycosylation pathways with similar polyisoprenol lipid carriers and topology (i.e., orientation across the cell membranes). Here, we provide the first systematic phylogenomic report on the polyisoprenol biosynthesis pathways in the three domains of life. This study shows that, whereas the last steps of the polyisoprenol biosynthesis are unique to the respective domain of life of which they are characteristic, the enzymes required for basic unsaturated polyisoprenol synthesis can be traced back to the respective last common ancestor of each of the three domains of life. As a result, regardless of the topology of the tree of life that may be considered, the most parsimonious hypothesis is that these enzymes were inherited in modern lineages from the cenancestor. This observation supports the presence of an enzymatic mechanism to synthesize unsaturated polyisoprenols in the cenancestor and, since these molecules are notorious lipid carriers in glycosylation pathways involved in the synthesis of a wide diversity of prokaryotic cell walls, it provides the first indirect evidence of the existence of a hypothetical unknown cell wall synthesis mechanism in the cenancestor.

## Introduction

Cells from the three domains of life (archaea, bacteria and eukaryotes) are bound by cell membranes of which the main lipid components are the phospholipids. At first, the strong chemical and biosynthetic dissimilarities that exist between the archaeal phospholipids and those from bacteria and eukaryotes triggered a hot debate about the existence of lipid membranes in the last common ancestor of extant organisms, namely the cenancestor or LUCA (Koga et al., 1998; Wächtershäuser, 2003; Martin & Russell, 2003; Peretó, López-García & Moreira, 2004). Later, new phylogenomic analyses inferred the presence of metabolic pathways responsible for the synthesis of most phospholipid components in the cenancestor (Peretó, López-García & Moreira, 2004; Lombard & Moreira, 2011; Lombard, López-García & Moreira, 2012a; Lombard, López-García & Moreira, 2012c). Therefore, the debates have progressively shifted from arguing the existence of membranes to discuss their composition and properties (Lane & Martin, 2012; Lombard, López-García & Moreira, 2012b; Sojo, Pomiankowski & Lane, 2014). Contrary to cell membranes, the early evolution of cell walls has been scarcely studied. Cell walls indeed provide essential structural support and external interactions in modern organisms (Albers & Meyer, 2011), but their extreme diversity makes the study of their early evolution very challenging.

Despite the stunning diversity that exists among prokaryotic cell envelopes, the synthesis of many of their main components (e.g., *N*-or *O*-glycosylated S-layer proteins, peptidoglycan, *O*-antigen LPS, teichoic acids, exopolysaccharides) relies on comparable glycosylation pathways (Hartley & Imperiali, 2012; Lombard, 2016). These pathways are all located in the cell membranes, are mediated by similar lipid carriers and have the same orientation across the membrane. Their respective mechanisms can be described in three major steps: (1) synthesis of an oligomer on a lipid carrier in the cytoplasmic side of the cell membranes; (2) “flipping” of the oligomer-linked lipid carrier to the opposite side of the membrane; and (3) oligomer transfer from the lipid carrier to the acceptor molecule (e.g., protein or external polymer). The eukaryotic protein *N*-glycosylation also meets these criteria, although it takes place in the ER membranes instead of the plasma membranes. The proteins involved in these glycosylation pathways belong to a small number of protein superfamilies, some of which could be traced back to the cenancestor (Lombard, 2016). The large diversity of the glycosylation pathways makes it difficult to untangle the specific evolutionary relationships that exist among them but, despite their differences, the prokaryotic cell wall synthesis mechanisms have at least one element in common: they all use polyisoprenol phosphate lipid carriers (Hartley & Imperiali, 2012).

**Figure 1:**
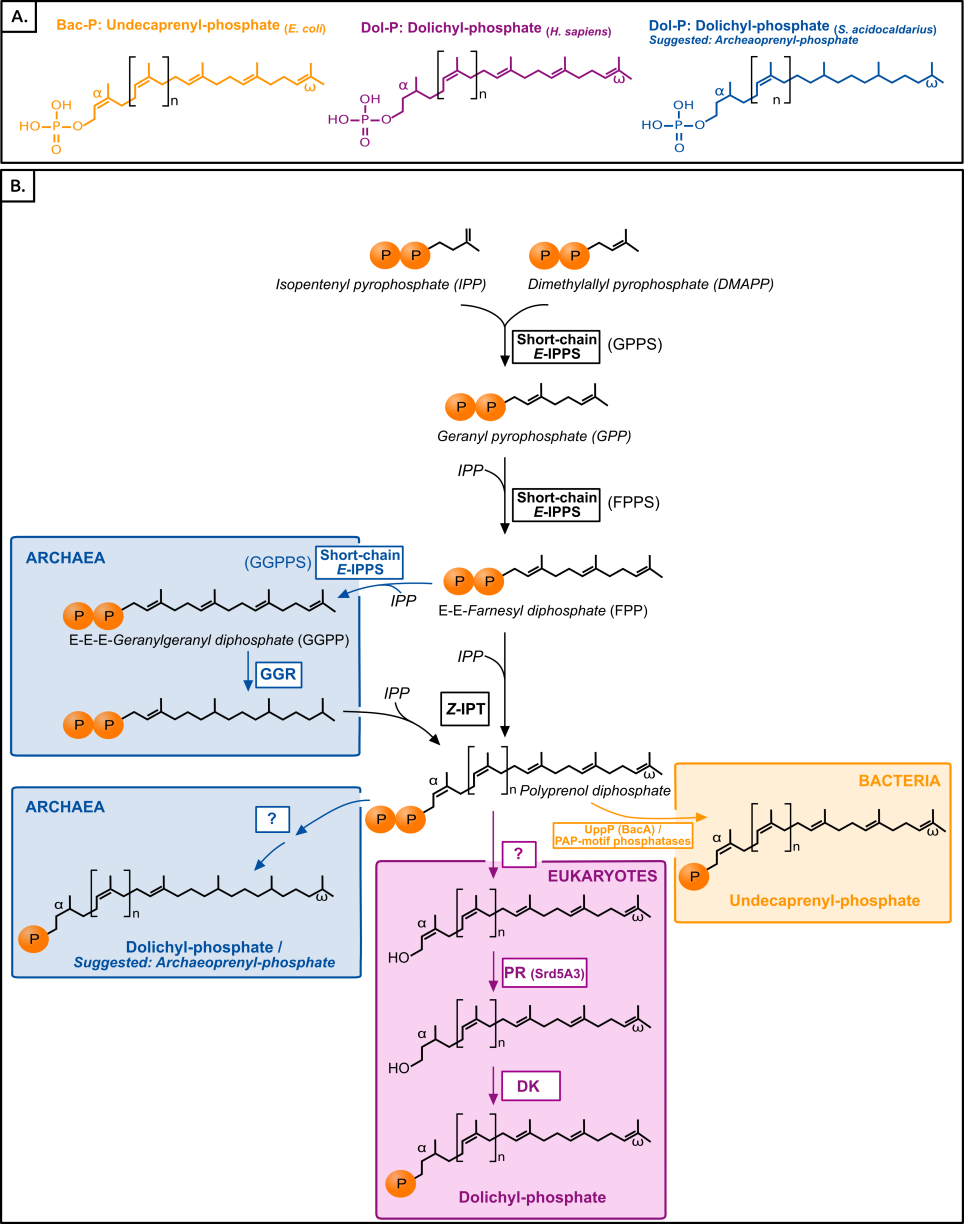
(A) Typical polyisoprenol lipid carriers in the three domains of life. (B) Polyisoprenol-phosphate biosynthesis pathways in the three domains of life. Steps outside boxes are common to all pathways, whereas steps in boxes are characteristic of only one domain of life. Unknown steps are labeled with a question mark. GPPS, geranyl diphosphate synthase; FPPS, farnesyl diphosphate synthase; GGPPS, geranylgeranyl diphosphate synthase; GGR, geranylgeranylreductase; IPT, isopentenyl transferase; UppP, undecaprenyl pyrophosphate phosphatase; PR, polyprenol reductase; DK, dolichol kinase.

Polyisoprenol phosphates are long unsaturated chains with a varying number of isoprene units and an *α*-terminal phosphate group (Fig. 1). The eukaryotic polyisoprenol phosphate is called dolichol-phosphate (Dol-P) and it is characterized by the saturation of its *α*-unit. Archaeal lipid carriers are *α*- and *ω*-polysaturated and also called Dol-P. Bacterial polyisoprenols are fully unsaturated and their names vary according to their length (Meyer & Albers, 2013), so they will be referred to as bactoprenol phosphate (Bac-P) for simplicity.

Similar to other isoprenoids, the polyisoprenols are initially synthesized through the sequential condensation of isopentenyl pyrophosphate (IPP) and allylic prenyl diphosphates, the first of which is dimethylallyl pyrophosphate (DMAPP, Fig. 1). The first 3–4 condensations in polyisoprenol biosynthesis require regular *E*-prenyltransferases (IPPS) but, contrary to other isoprenoids, later elongations involve *Z*-type links (Ogura & Koyama, 1998; Schenk, Fernandez & Waechter, 2001; Guan et al., 2011). The rest of the polyisoprenol phosphate synthesis pathways differs in each domain of life (Fig. 1). In bacteria, polyprenol diphosphate must be dephosphorylated into Bac-P. Most (75%) of this activity is catalyzed by an undecaprenyl pyrophosphate phosphatase (UppP/BacA) (El Ghachi et al., 2004; Chang et al., 2014; Manat et al., 2015), and complemented by the members of a promiscuous family of PAP-motif phosphatases (Stukey & Carman, 1997; El Ghachi et al., 2005; Touzé, Blanot & Mengin-Lecreulx, 2008). The involvement in this dephosphorylation step of other components, such as a supplementary cytoplasmic phosphatase or a polyprenol diphosphate translocation mechanism, has been suggested (see below), but their identity remains unknown. In archaea, the *ω*-terminal and internal saturated units are reduced by the geranylgeranyl reductases (GGR, Fig. 1) either before the elongation of FPP/GGPP into Dol-P or later on (Sato et al., 2008; Guan et al., 2011; Naparstek, Guan & Eichler, 2012). The rest of the archaeal Dol-P biosynthesis pathway remains unknown. Finally, in eukaryotes, polyprenol diphosphate is dephosphorylated, *α*-reduced (Cantagrel et al., 2010) and rephosphorylated (Cantagrel & Lefeber, 2011, Fig. 1). Here, a phylogenomic analysis of the proteins involved in the polyisoprenol phosphate biosynthesis pathways was performed in the three domains of life. This analysis provides promising insights into the unexplored question of the early evolution of cell walls.

## Material and Methods

The original sequence seeds were selected from KEGG (http://www.genome.jp/kegg/) and verified in the literature. BLASTp (Altschul et al., 1990) were carried out against a selection of representative genomes (Fig. S1) or the non-redundant (nr) dataset on GenBank (http://www.ncbi.nlm.nih.gov/Genbank), depending on results. When homologues were difficult to detect, more distant homologues were searched for using PSI-BLAST (Altschul et al., 1997) or HMM profiles and the hmmsearch tool implemented in the HMMER v3.1b2 webserver (http://hmmer.org/download.html and Finn et al., 2015). The alignments were carried out with MUSCLE v3.8.31 (Edgar, 2004) and trimmed with the program NET of the MUST package (Philippe, 1993). The preliminary phylogenies were reconstructed using FastTree v.2.1.7 (Price, Dehal & Arkin, 2010). Representative sequences were selected from these phylogenies to carry out more accurate analyses, and realigned using MUSCLE on the GUIDANCE server (http://guidance.tau.ac.il/ver2/, Penn et al., 2010). The final alignments are provided in Fig. S2 and were trimmed based on the statistical scores provided by GUIDANCE. The final phylogenies were constructed using MrBayes 3.2.6 (Ronquist et al., 2012) (LG substitution model (Le & Gascuel, 2008); 4 Γ categories; 4 chains of 1,000,000 generations; tree sampling every 100 generations; 25% generations discarded as “burn in”) and RaxML-HPC2 8.1.24 (Stamatakis, 2014) (LG +Γ model (Le, Dang & Gascuel, 2012); 4 rate categories; 100 bootstrap replicates) implemented in the CIPRES Science Gateway (Miller, Pfeiffer & Schwartz, 2010). Bayesian and maximum likelihood phylogenies gave comparable results.

## Results and Discussion

Despite being widespread in archaea, bacteria and eukaryotes, IPP and DMAPP are synthesized by respective pathways in each domain of life (Lombard & Moreira, 2011; Dellas et al., 2013; Vranová, Coman & Gruissem, 2013). A previous study on the evolution of these pathways concluded that the cenancestor had a primitive mevalonate pathway that could have synthesized IPP and DMAPP in this organism, and that each domain of life adopted its specific biosynthesis pathway later on (Lombard & Moreira, 2011). The *E*-type prenyl condensations are carried out by *E*-prenyltransferases (IPPS). The evolution of these proteins has been described in prokaryotes (Lombard, López-García & Moreira, 2012a) and the analysis is now extended to eukaryotes (see Fig. S3 for details). In summary, these analyses are not conclusive on the specific relationships that may exist among IPPS from the three domains of life, but they support the presence of IPPS homologues in the respective common ancestor of each domain of life. As a result, their presence can also be inferred in the cenancestor independently of the topology of the tree of life that may be favored.

**Figure 2:**
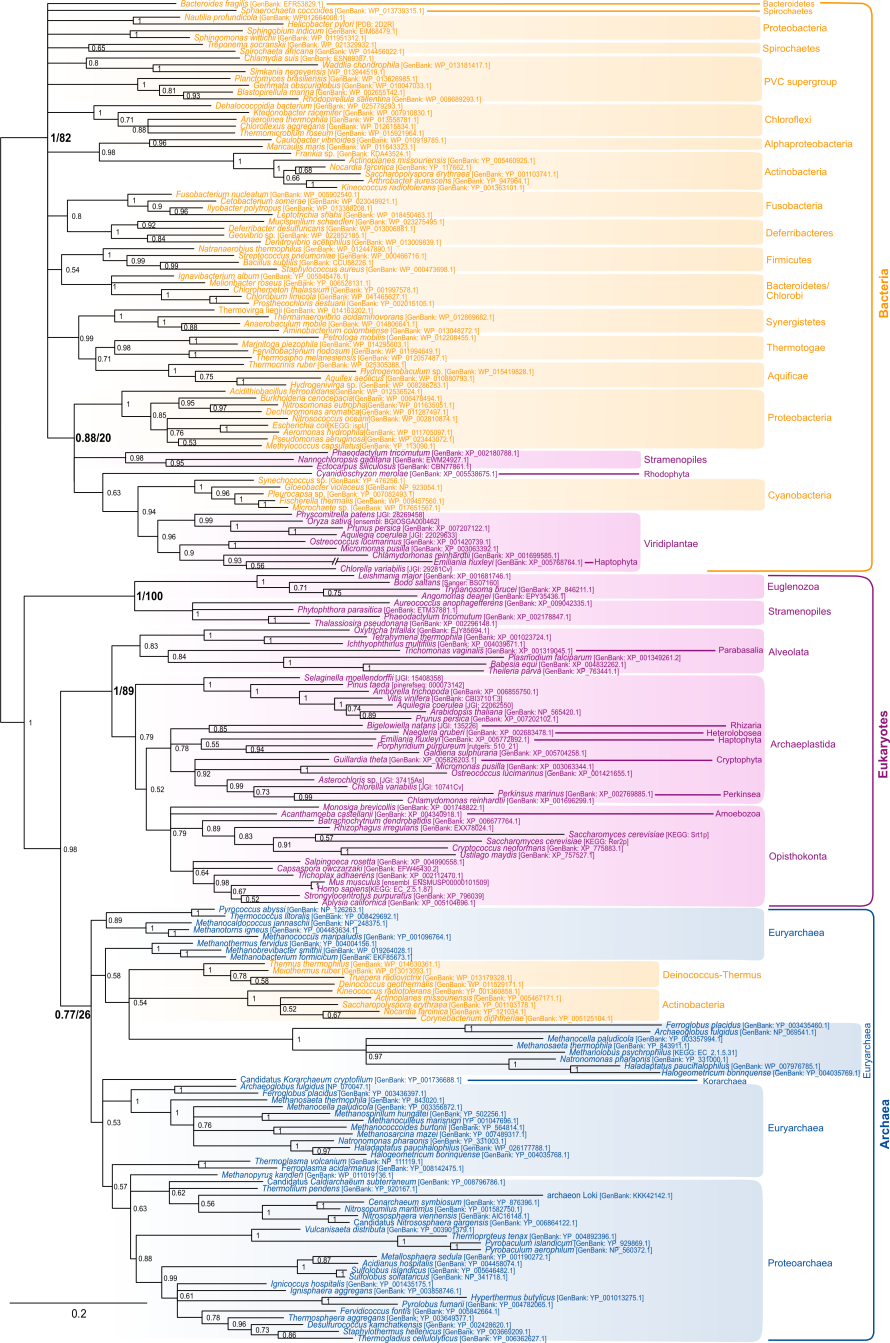
Bayesian phylogeny of the *Z*-IPTase homologues. The tree is unrooted and was reconstructed using 197 representative sequences and 202 conserved sites. Multifurcations correspond to branches with Bayesian posterior probabilities <0.5, whereas numbers at nodes indicate Bayesian posterior probabilities higher than 0.5. The bootstrap values from the maximum likelihood analyses have been reported on basal and major nodes. Colors of leaves represent the affiliation of sequences to their respective domain of life: archaea (blue), bacteria (orange) and eukaryotes (purple).

The most characteristic enzyme of the polyisoprenol phosphate biosynthesis pathway is the *Z*-isopentenyltransferase (*Z*-IPTase, Fig. 1) (Shimizu, Koyama & Ogura, 1998). The phylogeny of the *Z*-IPTases (Fig. 2) shows archaeal and bacterial monophyletic clades (Bayesian posterior probability (BPP) = 0.77 and BPP = 1, respectively) and several groups of eukaryotic sequences. The internal relationships within the archaeal clade are poorly resolved, which is not unexpected for a single-gene tree. The euryarchaea are paraphyleticand contain a group made up of distant paralogues and bacterial sequences probably acquired through horizontal gene transfer (HGT). Nevertheless, *Z*-IPTase homologues are widespread among archaea and the monophyly of the archaeal and proteoarchaeal (TACK superphylum, Petitjean et al., 2015) sequences support the presence of this gene in the Last Archaeal Common Ancestor. The deep phylogenetic relationships within the bacterial clade are also unresolved. Yet, the wide diversity of bacteria in which *Z*-IPTase homologues may be detected, together with the fact that most bacterial sequences group according to their taxonomic classification supports the ancestral presence of this gene in bacteria. Some polyphyletic sequences from plastid-bearing eukaryotes branch within a group of cyanobacteria and proteobacteria (BPP = 0.88) and likely have plastidial ancestors. The rest of the eukaryotic sequences are paraphyletic. The largest eukaryotic clade (BPP = 1) is made up of diverse sequences that cluster according to major eukaryotic taxa (e.g., opisthokonts, streptophytes, alveolates). Some internal branching is suggestive of inter-eukaryotic HGT, such as in organisms known to have had secondary plastid endosymbioses, which branch among archaeplastida. A smaller cluster of divergent euglenozoan and stramenopile sequences (BPP = 1) forms a sister group to the large eukaryotic/archaeal clade. Some of euglenozoa are known to synthesize shorter Dol-P molecules than other eukaryotes, so it is possible that the phylogenetic position of these sequences reflects a reconstruction artifact due to their high divergence (Schenk, Fernandez & Waechter, 2001). Ultimately, if we accept that the few paraphylies and polyphylies in this tree result from reconstruction artifacts due to the extreme sequence divergence of *Z*-IPTases across the three domains of life, this (unrooted) phylogeny supports the presence of *Z*-IPTases in the respective last common ancestors of each domain of life and, regardless of the topology of tree of life that may be invoked, in the cenancestor.

The following steps in the polyisoprenol phosphate biosynthesis pathways are specific to each domain of life (Fig. 1). In bacteria, the polyprenol diphosphate is dephosphorylated into Bac-P by UppP (BacA) or a family of periplasmic PAP-motif phosphatases. UppP and the PAP-motif phosphatases are not evolutionary related to each other and, until recently, it was thought that they were respectively responsible for the cytoplasmic *de novo* synthesis or the periplasmic recycling of polyprenol diphosphate (Bickford & Nick, 2013; Manat et al., 2014). The recent discovery that they all function in the periplasmic side of the membrane implies that the *de novo* polyprenol diphosphate dephosphorylation requires either the activity of an unknown cytoplasmic phosphatase, or the translocation to the periplasmic side of the membrane through an unknown mechanism (Chang et al., 2014; Manat et al., 2015; Teo & Roper, 2015). Although some candidates have been suggested to mediate the polyprenol (di)phosphate translocation (Sanyal & Menon, 2010; Chang et al., 2014; Manat et al., 2015), these hypotheses require biochemical verification. Thus, only the two known phosphatase families will be studied here.

UppP homologues were detected in archaea and a wide diversity of bacteria but not in eukaryotes, even when HMM searches for distantly related sequences were carried out (see ‘Methods’). Since relatively little is known about the last steps in archaeal polyisoprenol biosynthesis, the possible participation of these archaeal homologues in this pathway seems worth testing experimentally. The phylogeny of the UppP homologues is discussed in more detail in Fig. S4 but, in summary, the respective monophyly of archaeal and bacterial UppP homologues (BPP = 0.96) suggests that this gene could have been present in the respective ancestors of both archaea and bacteria and, therefore, in the cenancestor. Importantly, this suggests that the cenancestor could have had a minimum set of proteins representative of all the enzymes currently known to be required to synthesize not only polyisoprenol chains but also functional lipid carriers (Bac-P). Regarding the PAP-motif phosphatases, several representatives of the family have been biochemically characterized (Fernández & Rush, 2001; El Ghachi et al., 2005; Manabe et al., 2009) and their homologues are widespread in the three domains of life, but their phylogeny is poorly resolved and dominated by the presence of paralogues and xenologues (see Fig. S5 for details). As a result, it is not possible at this stage to discriminate between a cenancestral or more recent origin of the PAP-motif phosphatases.

In archaea, the *ω*-terminal and internal units are reduced by the GGRs (Fig. 1). A recent phylogenomic analysis of the GGRs suggested (1) the ancestral presence of this gene in archaea, followed by a complicated history of duplications and HGTs; (2) an unclear origin for these genes in bacteria; and (3) a likely plastidial origin of these genes in eukaryotes (Lombard, López-García & Moreira, 2012a); thus, the presence of GGRs in the cenancestor is not supported. The rest of the archaeal Dol-P biosynthesis pathway remains uncharacterized, so particular attention was drawn to the possible archaeal homologues of the enzymes in the eukaryotic Dol-P synthesis pathway.

The specific steps in the eukaryotic pathway consist in the dephosphorylation, *α*-reduction and rephosphorylation of the polyprenol diphosphate (Fig. 1). The eukaryotic polyprenol diphosphate phosphatase is unknown (Cantagrel & Lefeber, 2011; Bickford & Nick, 2013). Sequences similar to the polyisoprenol *α*-unit reductases (PR, Fig. 1) are widespread in eukaryotes but they are absent in archaea and rare in bacteria (these are likely xenologues). This implies that archaea use an alternative way to saturate the *α*-unit of their Dol-P. The PR phylogeny (see Fig. S6 for details) suggests that this protein is an early eukaryotic innovation that was developed before the Last Eukaryotic Common Ancestor and duplicated later in several eukaryotic lineages. Finally, homologues of the dolichol kinase (DK) were easily detected among a large diversity of eukaryotes, but only in a few prokaryotes. PSI-BLAST searches (Altschul et al., 1997) revealed a distant evolutionary relationship between the DKs and the CDP-diacylglycerol synthases (CdsA) involved in phospholipid synthesis (Sparrow & Raetz, 1985). The evolution of both functions was studied (Fig. S7) but the quality of the sequence alignment was very poor, the resulting phylogenies are unreliable and so are the conclusions that can be taken from them. This analysis, however, questions the existence of DK functional orthologues in most prokaryotes, especially in Euryarchaea. The lack of homologues of the last steps of eukaryotic Dol-P synthesis in most archaeal genomes raises the question of how these organisms finish the synthesis of their polyisoprenol phosphate lipid carriers.

**Figure 3:**
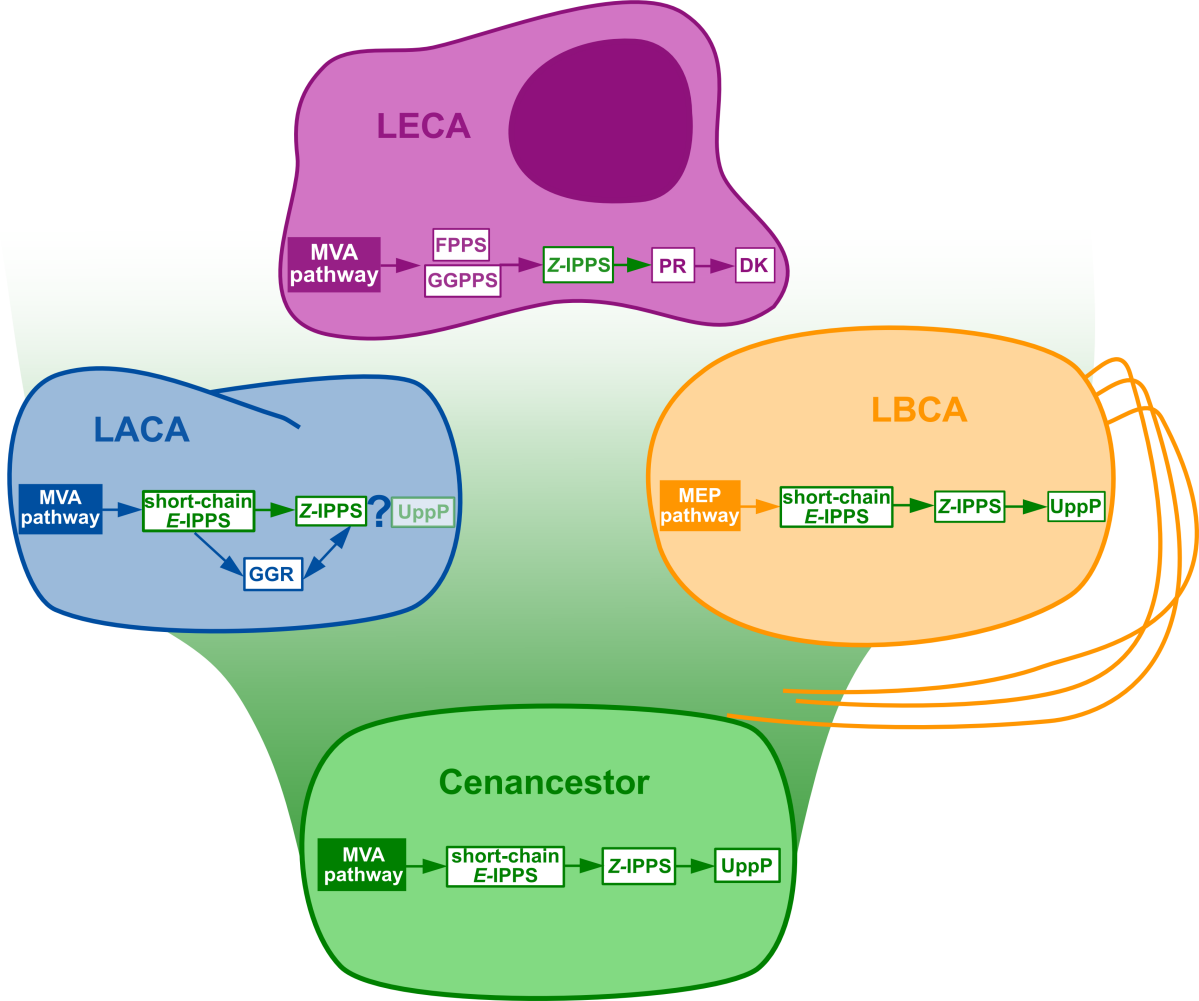
Summary of the phylogenomic results of the enzymes involved in the polyisoprenol biosynthesis pathways. Only known genes are presented, so the eukaryotic and archaeal pathways are not complete. MVA, mevalonate; MEP, Methylerythritol phosphate; LACA, Last Archaeal Common Ancestor; LBCA, Last Bacterial Common Ancestor; LECA, Last Eukaryotic Common Ancestor. Refer to Fig. 1 for other abbreviations.

## Conclusions

The initial metabolic steps of the polyisoprenol biosynthesis pathways (from IPP/DMAPP synthesis to polyprenol diphosphate) are widespread in the three domains of life, and their presence can be inferred in at least the respective ancestor of each prokaryotic domain of life (Fig. 3). The later steps differ in each domain. Bacterial UppP (BacA) may be ancestral to both bacteria and archaea, but the evolution of the PAP-motif phosphatases is less clear. PR, and probably DK as well, are eukaryotic innovations. Archaea use their ancestral GGR, they do not have PR homologues and most of them lack DK homologues. Although neither the archaeal nor the eukaryotic pathways are fully described yet, the available information suggests that these domains have different means of synthesizing their polyisoprenol phosphates. This is congruent with the fact that, despite having the same name, eukaryotic Dol-P and archaeal Dol-P are chemically different from each other (Fig. 1). In order to avoid any confusion, we propose the archaeal polysaturated polyisoprenols (Guan et al., 2010; Guan et al., 2011; Chang et al., 2015) to be called “archaeoprenols” from now on.

Polyprenol diphosphate biosynthesis is ancestral to each domain of life and, therefore, the most parsimonious hypothesis to explain its distribution is that it was inherited from the cenancestor. Although the mechanism that provides this molecule to the periplasmic phosphatases remains unknown (Chang et al., 2014; Manat et al., 2015), the possibility that UppP homologues may also be ancestral to both prokaryotic domains similarly suggests that the cenancestor could even have been able to synthesize some sort of Bac-P. Polyisoprenols may carry out pleiotropic functions (Cantagrel & Lefeber, 2011; Hartley & Imperiali, 2012), but their most notorious role is as lipid carriers in a number of glycosylation pathways (Lombard, 2016). In prokaryotes, these glycosylations are systematically related to the synthesis of cell wall components, so it is reasonable to postulate that hypothetical cenancestral polyisoprenols may have played the role of lipid carriers in early glycosylation mechanisms, possibly related to cell wall synthesis. Yet, this hypothesis will need to be updated when more information about the polyisoprenol biosynthesis pathways will be available.

Although the presence of a polyisoprenol phosphate biosynthesis pathway in the cenancestor is a promising piece of evidence to support the existence of cell walls in this organism, the large diversity of microbial cell walls remains an obstacle to risk a hypothesis on the specific nature of this cenancestral structure. Interestingly, the presence of at least two promising glycosyltransferase superfamilies (HPT and MurG) has been inferred in the cenancestor (Lombard, 2016). The proteins in these families are responsible for the transfer of soluble monosaccharides to polyisoprenol phosphate lipid carriers in bacterial peptidoglycan synthesis (Mohammadi et al., 2007) and archaeal S-layer protein *N*-glycosylation (Meyer & Albers, 2013). If we sum up the results presented here and those about glycosyltransferases (Lombard, 2016), it is tempting to say that the cenancestor could have been able to use its HPT and MurG homologues to synthesize an oligosaccharide on a Bac-P lipid carrier. The question that remains open is the final acceptor of that hypothetical cenancestral oligosaccharide, as that is the element that would define the nature of the putative cenancestral cell wall. One way to tackle this question would be studying the transferases that convey the glycans from the lipid carrier to various donors. These transferases were phylogenomically analyzed in (Lombard, 2016), but none of them revealed a widespread function that would provide an obvious candidate to make up a particular type of cell wall in the cenancestor. For instance, S-layers have been put forward as possible first cell wall components (Albers & Meyer, 2011; Fagan & Fairweather, 2014). The protein *N*-glycosylation oligosaccharyltransferases and *O*-mannosylation transferases responsible for S-layer protein glycosylation are indeed probably ancestral to eukaryotes and archaea, but their ancestral presence in bacteria is uncertain (Lombard, 2016). Therefore, the presence of glycosylated S-layers proteins in the cenancestor remains inconclusive.

It has been argued that the strong conservation of polyisoprenol phosphates as lipid carriers may be functional: they may play important regulatory roles on glycan mechanisms, their biochemistry may be particularly adapted to glycan translocation across phospholipid bilayers or they can be beneficial to the formation of large protein complexes with which they frequently interact (Hartley & Imperiali, 2012). Contrary to these possible strong pressures to maintain polyisoprenol phosphates as lipid carriers, the difficulty to determine the final acceptor of the hypothetical cenancestral oligosaccharide may be explained by the likely strong selective pressures that acted on the exposed cell wall components to diversify. For instance, one specific type of cell wall may have actually existed in the cenancestor, but the strong selective pressures exerted on the cell wall glycocalyx may have resulted in the independent replacement of the original cell wall in many lineages. Another possibility is that the cenancestral cell wall glycosylation was promiscuous and that specific cell wall types only evolved later in modern lineages, following the same selective pressures. We are at a time of dynamic characterization of prokaryotic envelopes and comparative studies, so new evidence may soon shed light on more mechanistic similarities among cell wall syntheses in the three domains of life and complement our knowledge of the cenancestral envelopes.

##  Supplemental Information

10.7717/peerj.2626/supp-1 Figure S1 List of the genomes used in this analysis

10.7717/peerj.2626/supp-2 Figure S2 Multiple alignments of the genes presented in this work

10.7717/peerj.2626/supp-3 Figure S3 Bayesian phylogeny of the E-isoprenytransferases (IPPS)

10.7717/peerj.2626/supp-4 Figure S4 Bayesian phylogeny of the undecaprenyl pyrophosphate phosphatase (UppP)

10.7717/peerj.2626/supp-5 Figure S5 Bayesian phylogeny of the PAP-motif phosphatases

10.7717/peerj.2626/supp-6 Figure S6 Polyprenol reductase phylogenies

10.7717/peerj.2626/supp-7 Figure S7 FastTree phylogeny of the dolichol kinases

## Additional Information and Declarations

### Data Availability

The following information was supplied regarding data availability: The raw data has been supplied as Supplementary File.
