# Supplementary material for: Early evolution of polyisoprenol biosynthesis and the origin of cell walls
Source: PeerJ. 2016 Oct 26;4:e2626. doi: 10.7717/peerj.2626 (PMC5088576; doi:10.7717/peerj.2626)
Supplement: Figure S1 [file peerj-04-2626-s001.pdf]

Suppl. Figure 1. List of the genomes used in this analysis.

<u>Eukaryotes</u>

Acanthamoeba castellanii
Allomyces macrogynus
Amborella trichopoda
Amphimedon queenslandica
Angomonas deanei
Aplysia californica
Aquilegia coerulea
Arabidopsis thaliana
Asterochloris sp. Cgr/DA1phos
Aureococcus anophagefferens
Babesia equi
Bathycoccus prasinos
Batrachochytrium dendrobatidis JAM81
Bigelowiella natans
Blastocystis hominis
Bodo saltans
Calliarthron tuberculosum
Capsaspora owczarzaki
Chlamydomonas reinhardtii
Chlorella variabilis
Chondrus crispus
Cryptococcus neoformans
Cyanidioschyzon merolae
Cyanophora paradoxa
Dictyostelium discoideum
Drosophila melagonaster
Emiliania huxleyi CCMP1516
Entamoeba histolytica
Fonticula alba
Galdieria sulphuraria
Giardia lamblia
Gregarina niphandrodes
Guillardia theta CCMP2712
Hydra magnipapillata
Ichthyophthirius multifiliis
Klebsormidium flaccidium
Leishmania major
Micromonas pusilla CCMP1545
Mnemiopsis leidyi
Monosiga brevicollis
Mus musculus
Naegleria gruberi
Oryza sativa
Ostreococcus lucimarinus
Oxytricha trifallax
Paramecium tetraurelia
Perkinsus marinus
Phaeodactylum tricornutum
Physcomitrella patens
Phytophthora parasitica
Pinus taeda
Plasmodium falciparum
Polysphondylium pallidum
Porphyridium purpureum
Prunus persica
Pyropia yezoensis
Reticulomyxa filosa
Rhizophagus irregularis DAOM 181602
Saccharomyces cerevisiae
Salpingoeca rosetta
Schizochytrium aggregatum ATCC 28209
Schizosaccharomyces pombe
Selaginella moellendorffii
Spironucleus salmonicida
Spizellomyces punctatus DAOM BR117
Strongylocentrotus purpuratus
Symbiodinium minutum
Tetrahymena thermophila
Thecamonas trahens ATCC 50062
Theileria annulata
Toxoplasma gondii
Trichomonas vaginalis G3
Trichoplax adhaerens
Trypanosoma vivax
Ustilago maydis
Volvox carteri

<u>Archaea</u>

Acidianus hospitalis W1
Acidilobus saccharovorans 345-15
Aeropyrum pernix K1
Aigarchaeota archaeon JGI 0000001-A7
Archaeoglobus fulgidus DSM 4304
Caldisphaera lagunensis DSM 15908
Caldivirga maquilingensis IC-167
Candidatus *Aenigmarchaeum subterraneum*

Candidatus *Caldiarchaeum subterraneum*
Candidatus *Haloredivivus* sp. G17
Candidatus *Iainarchaeum andersonii*
Candidatus *Korarchaeum cryptofilum* OPF8
Candidatus *Nanosalina* sp. J07AB43
Candidatus *Nitrososphaera gargensis* Ga9.2
Candidatus *Parvarchaeum acidiphilum* ARMAN-4_'5-way FS'
Candidatus *Parvarchaeum acidophilus* ARMAN-5_'5-way FS'
Cenarchaeum symbiosum A
Desulfurococcus kamchatkensis 1221n
Ferroglobus placidus DSM 10642
Ferroplasma acidarmanus fer1
Fervidicoccus fontis Kam940
Haladaptatus paucihalophilus
Haloferax volcanii DS2
Halogeometricum borinquense DSM 11551
Hyperthermus butylicus DSM 5456
Ignicoccus hospitalis KIN4/I
Ignisphaera aggregans DSM 17230
Metallosphaera sedula DSM 5348
Methanobacterium formicicum DSM 3637
Methanobrevibacter arboriphilus ANOR1
Methanobrevibacter smithii
Methanocaldococcus fervens AG86
Methanocaldococcus jannaschii
Methanocella paludicola SANAE
Methanococcoides burtonii
Methanococcus maripaludis C5
Methanoculleus marisnigri JR1
Methanopyrus kandleri
Methanosaeta thermophila
Methanosarcina mazei Tuc01
Methanospirillum hungatei
Methanotorris igneus
Methanothermococcus thermolithotrophicus DSM 2095
Methanothermus fervidus DSM 2088
Nanoarchaeum equitans Kin4-M
Natronomonas pharaonis DSM 2160
Nitrosopumilus maritimus SCM1
Nitrososphaera viennensis
Pyrobaculum aerophilum str. IM2
Pyrobaculum islandicum DSM 4184
Pyrococcus abyssi GE5
Pyrolobus fumarii 1A
Staphylothermus hellenicus
Staphylothermus marinus F1
Sulfolobus islandicus HVE10/4
Sulfolobus solfataricus P2
Sulfolobus tokodaii
Thermococcus kodakarensis KOD1
Thermococcus litoralis
Thermofilum pendens Hrk 5
Thermogladius cellulolyticus 1633
Thermoplasma volcanium GSS1
Thermoproteus tenax Kra 1
Thermosphaera aggregans DSM 11486
Vulcanisaeta distributa DSM 14429

<u>Bacteria</u>

Acaryochloris sp. CCMEE 5410
Acetobacter nitrogenifigens DSM 23921
Acholeplasma granularum ATCC 19168
Acidimicrobium ferrooxidans DSM 10331
Acidithiobacillus ferrooxidans ATCC 23270
Acidobacterium capsulatum ATCC 51196
Actinobaculum massiliae ACS-171-V-Col2
Actinoplanes friuliensis DSM 7358
Actinoplanes missouriensis 431
Actinopolyspora halophila DSM 43834
Aeromonas hydrophila 116
Agarivorans albus
Akkermansia muciniphila ATCC BAA-835
Alcanivorax pacificus W11-5
Alcanivorax sp. DG881
Algoriphagus marincola HL-49
Alicyclobacillus acidocaldarius DSM 446
Aminomonas paucivorans DSM 12260
Ammonifex degensii KC4
Anaerolinea thermophila UNI-1
Anaplasma phagocytophilum str. Dog2
Anoxybacillus kamchatkensis G10
Aquifex aeolicus VF5
Armatimonadetes bacterium
Arthrobacter aurescens TC1
Arthrospira platensis C1
Aurantimonas coralicida DSM 14790
Azoarcus sp. BH72
Bacillus subtilis B7-s
Bacteroides fragilis HMW 610
Bdellovibrio bacteriovorus HD100
Bifidobacterium bifidum BGN4

Blastopirellula marina DSM 3645
Bordetella avium 197N
Bordetella bronchiseptica 7E71
Borrelia burgdorferi 118a
Brachyspira innocens ATCC 29796
Brevibacterium album DSM 18261
Burkholderia cenocepacia
Caldilinea aerophila DSM 14535 = NBRC 104270
Caldisericum exile AZM16c01
Calditerrivibrio nitroreducens DSM 19672
Caldithrix abyssi DSM 13497
Campylobacter gracilis
Campylobacter jejuni 10186
Candidatus *Aerophobus profundus*
Candidatus *Aminicenans sakinawicola* JGI OTU-1
Candidatus *Caldatribacterium californiense*
Candidatus *Calescibacterium nevadense* OTU 1
Candidatus *Fervidibacter sacchari* JGI OTU-1
Candidatus *Nitrospira defluvii*
Caulobacter crescentus NA1000
Chlamydia pneumoniae B21
Chlamydophila felis Fe/C-56
Chlorobium limicola
Chlorobium luteolum DSM 273
Chlorobium tepidum TLS
Chloroflexus aurantiacus J-10-fl
Chlorogloeopsis fritschii PCC 6912
Chloroherpeton thalassium ATCC 35110
Chroococcidiopsis thermalis PCC 7203
Chrysiogenes arsenatis DSM 11915
Chthoniobacter flavus Ellin428
Chthonomonas calidirosea T49
Clostridium acetobutylicum ATCC 824
Clostridium aerotolerans DSM 5434
Clostridium saccharobutylicum
Collinsella tanakaei
Conexibacter woesei
Coprobacillus sp. 3_3_56FAA
Coprothermobacter proteolyticus DSM 5265
Corynebacterium diphtheriae 241
Cytophaga aurantiaca DSM 3654
Cytophaga hutchinsonii ATCC 33406
Dehalococcoides mccartyi 195
Dehalogenimonas lykanthroporepellens BL-DC-9
Deinococcus deserti VCD115
Deinococcus radiodurans R1
Desulfarculus baarsii DSM 2075
Desulfatibacillum alkenivorans AK-01
Desulfobacter curvatus DSM 3379
Desulfovibrio alkalitolerans DSM 16529
Desulfurispirillum indicum S5
Desulfurobacterium thermolithotrophum
Desulfovibrio acrylicus
Dethiosulfovibrio peptidovorans DSM 11002
Dichelobacter nodosus VCS1703A
Dictyoglomus thermophilum H-6-12
Elusimicrobium minutum Pei191
Enterococcus faecalis 62
Entomoplasma lucivorax ATCC 49196
Escherichia coli K2
Ferrovum myxofaciens
Fervidobacterium nodosum Rt17-B1
Fibrobacter succinogenes S85
Fimbriimonas ginsengisoli Gsoil 348
Fischerella muscicola PCC 7414
Fischerella thermalis
Flavobacterium antarcticum DSM 19726
Frankia alni ACN14a
Frankia sp. BMG5.23
Fusobacterium nucleatum
Fusobacterium periodonticum ATCC 33693
Gallionella capsiferriformans ES-2
Gemmata obscuriglobus UQM 2246
Gemmatimonadetes bacterium KBS708
Gemmatimonas aurantiaca T-27
Geobacter sulfurreducens PCA
Geovibrio sp. L21-Ace-BES
Gloeobacter violaceus PCC 7421
Gloeocapsa sp. PCC 73106
Glycomyces arizonensis DSM 44726
Halanaerobium praevalens DSM 2228
Halobacteroides halobius DSM 5150
Halothermothrix orenii
Helicobacter pylori 35A
Herpetosiphon aurantiacus DSM 785
Hydrogenivirga sp. 128-5-R1-1
Hydrogenobacter thermophilus TK-6
Hydrogenobaculum sp. SN
Hyphomonas sp. 25B14_1
Ignavibacterium album JCM 16511
Ilumatobacter coccineus
Ilyobacter polytropus
Isosphaera pallida ATCC 43644
Jiangella gansuensis DSM 44835
Kandleria vitulina DSM 20405
Kiloniella laminariae DSM 19542
Kineococcus radiotolerans ATCC BAA-149
Kitasatospora setae KM-6054

Kitasatospora sp. NRRL B-11411
Kordiimonas gwangyangensis JCM 12864
Kosmotoga olearia TBF 19.5.1
Ktedonobacter racemifer DSM 44963
Lactobacillus acidophilus ATCC 4796
Legionella pneumophila Leg01/16
Lentisphaera araneosa HTCC2155
Leptospira sp. B5-022
Leptospirillum sp. Group I
Leptotrichia buccalis C-1013-b
Leptotrichia wadei
Lyngbya aestuarii BL J
Marinimicrobia bacterium JGI 0000039-D08
Marinithermus hydrothermalis DSM 14884
Marinitoga piezophila KA3
Mariprofundus ferrooxydans PV-1
Meiothermus ruber H328
Meiothermus silvanus DSM 9946
Melioribacter roseus P3M-2
Mesorhizobium australicum
Mesotoga prima MesG1.Ag.4.2
Methylobacillus glycogenes JCM 2850
Methylophilus methylotrophus
Microchaete sp. PCC 7126
Mitsuokella multacida DSM 20544
Mycoplasma alkalescens 14918
Myxococcus xanthus DK 1622
Natranaerobius thermophilus JW/NM-WN-LF
Nautilia profundicola AmH
Neisseria bacilliformis ATCC BAA-1200
Neisseria gonorrhoeae 1291
Neisseria meningiditis
Neochlamydia sp. S13
Nitratifractor salsuginis DSM 16511
Nitratiruptor sp. SB155-2
Nitrolancea hollandica Lb
Nitrosococcus halophilus Nc 4
Nitrosomonas eutropha C91
Nitrospina gracilis 3/211
Nocardia farcinica IFM 10152
Nocardiopsis alkaliphila YIM 80379
Nostoc punctiforme PCC 73102
Oceanimonas sp. GK1
Oceanithermus profundus DSM 14977
Opitutus terrae PB90-1
Parvularcula bermudensis HTCC2503
Pasteurella multocida 1500C
Pedobacter agri PB92
Persephonella marina EX-H1
Petrotoga mobilis SJ95
Phycisphaera mikurensis NBRC 102666
Pirellula staleyi DSM 6068
Planctomyces limnophilus DSM 3776
Planctomyces maris
Pleurocapsa sp. PCC 7327
Poribacteria bacterium WGA-3G
Prevotella aurantiaca JCM 15754
Propionibacterium acidipropionici DSM 4900
Prosthecochloris aestuarii DSM 271
Pseudomonas aeruginosa
Pseudomonas alcaliphila 34
Pseudonocardia autotrophica
Psychrobacter arcticus 273-4
Rhodopirellula baltica SH 1
Rhodospirillum centenum SW
Rickettsia felis URRWXCal2
Saccharopolyspora erythraea NRRL 2338
Salinimonas chungwhensis DSM 16280
Sebaldella termitidis
Simkania negevensis Z
Singulisphaera acidiphila DSM 18658
Sorangium cellulosum So0157-2
Sphingobium chlorophenolicum L-1
Sphingobium indicum
Sphingobium sp. AP49
Spirochaeta sp.
Spirochaeta thermophila DSM 6192
Staphylococcus aureus 1484-9
Streptococcus agalactiae 515
Streptomyces aurantiacus
Sulfurihydrogenibium subterraneum DSM 15120
Sulfurimonas autotrophica DSM 16294
Sulfurovum sp. AR
Synechococcus elongatus PCC 6301
Synechococcus sp. JA-3-3Ab
Synergistes jonesii
Synergistes sp. 3_1_syn1
Terrimonas ferruginea DSM 30193
Thermodesulfatator atlanticus DSM 21156
Thermodesulfobacterium thermophilum DSM 1276
Thermodesulfovibrio islandicus DSM 12570
Thermomicrobium roseum DSM 5159
Thermosipho africanus TCF52B
Thermotoga maritima MSB8
Thermotoga profunda
Thermotoga sp. A7A
Thermovibrio ammonificans HB-1
Thermovirga lienii

Thermus aquaticus Y51MC23
Thermus islandicus DSM 21543
Thiobacillus thioparus DSM 505
Treponema bryantii NK4A124
Treponema denticola
Truepera radiovictrix DSM 17093
Waddlia chondrophila WSU 86-1044
Xanthomonas oryzae ATCC 35933
Zavarzinella formosa DSM 19928
