# Supplementary material for: Early evolution of polyisoprenol biosynthesis and the origin of cell walls
Source: PeerJ. 2016 Oct 26;4:e2626. doi: 10.7717/peerj.2626 (PMC5088576; doi:10.7717/peerj.2626)

Suppl. Figure 3. Bayesian phylogeny of the *E*-isoprenytransferases (IPPS). Tree is unrooted and reconstructed using 208 sequences and 201 conserved sites. Multifurcations correspond to branches with Bayesian posterior probabilities <0.5, whereas numbers at nodes indicate Bayesian posterior probabilities higher than 0.5. The bootstrap values from the maximum likelihood analyses have been reported on basal and major nodes. Colors on leaves represent the affiliation of sequences to their respective domain of life: archaea (blue), bacteria (orange) and eukaryotes (purple).

IPPS are classified according to the size of their products into short-chain IPPS (up to ~5 isoprenoid units) and long-chain IPPS (>5 isoprenoid units). Short-chain and long-chain IPPS are homologous to each other but the size of the molecules they produce is constrained by differences in their substrate-binding pockets (Kellogg & Poulter, 1997). Point mutations may easily change the size of these pockets (Ohnuma et al., 1996, 1998), so substrate specificity for particular genes cannot be predicted from sequence analysis only and requires biochemical confirmation (Chen, Kroon & Poulter, 1994; Tachibana et al., 2000). Yet, previous phylogenetic analyses have shown that homologues of the IPPS family actually split in two clades that roughly correspond to short-chain and long-chain IPPS (Boucher, Kamekura & Doolittle, 2004; Lombard, López-García & Moreira, 2012). Both IPPS sub-families were shown to be probably ancestral to archaea and bacteria and, therefore, it is likely that they were present in the cenancestor (Lombard, López-García & Moreira, 2012). The evolution of the short-chain IPPS–including the FPP synthase (FPPS) and the GGPP synthase (GGPPS)–was reexamined here in order to also include the eukaryotes.

A preliminary phylogenetic tree was built to discriminate short- and long-chain IPPS (data not shown). The subsequent phylogeny limited to the short-chain IPPS (this figure) has three parts: 1) a large group containing most eukaryotic sequences (BPP = 0.68); 2) a mainly bacterial group at the bottom of the figure (BPP = 0.53); and 3) several unresolved paraphyletic archaeal groups, also containing a few bacterial sequences. Most bacterial and archaeal sequences group together according to their taxonomic classification, but the lack of strong phylogenetic signal prevents from obtaining a resolved phylogeny, especially among the archaeal section of the tree. A reason that may explain these resolution issues is the noise introduced by the very divergent eukaryotic sequences, which was less dominant in prokaryotic-only analyses published elsewhere (Lombard, López-García & Moreira, 2012).

Regarding the eukaryotic sequences, three groups are discernible: one group of plastid-bearing eukaryotes related to cyanobacteria in the bacterial clade (BPP = 1)–and, therefore, probably acquired

from the cyanobacterial ancestor of plastids; and two divergent eukaryotic paralogues (BPP = 1 in both cases). The eukaryotic paralogues can respectively be assigned to the FPPS and GGPPS functions. Both paralogues are widespread and likely ancestral to eukaryotes. Both paralogues are also related to a few prokaryotic basal sequences. The basal sequences related to the GGPPS are a small group of mixed archaea, whereas the basal prokaryotic sequences related to FPPS are a mix of cyanobacteria and Deinococcus-Thermus bacteria. The little number of basal prokaryotic sequences, their taxonomic diversity and the strong divergence of the eukaryotic sequences with regard to the other prokaryotic sequences suggest that the placement of these prokaryotic basal sequences could result from reconstruction artifacts and can hardly be seen as significant. The fact that the two short-chain IPPS paralogues are only widespread in eukaryotes argues in favor of the idea that short-IPPS were duplicated in the eukaryotic lineage prior to LECA. This phylogeny does not allow to confidently ascertain the origin of the eukaryotic short-chain IPPS, but it supports the monophyly of the eukaryotic sequences. Since the presence of IPPS genes can be traced back to the last common ancestor of eukaryotes (this work) and the respective common ancestors of bacteria and archaea (Lombard, López-García & Moreira, 2012), an IPPS gene is likely to have existed in the cenancestor.

Boucher Y, Kamekura M, Doolittle WF. 2004. Origins and evolution of isoprenoid lipid biosynthesis in archaea. *Molecular Microbiology.* 52:515–27. DOI: 10.1111/j.1365-2958.2004.03992.x.

Chen A, Kroon PA, Poulter C. 1994. Isoprenyl diphosphate synthases: Protein sequences comparisons, a phylogenetic tree and predictions of secondary structure. *Protein Science.* 3:600–607.

Kellogg BA, Poulter CD. 1997. Chain elongation in the isoprenoid biosynthetic pathway. *Current Opinion in Chemical Biology.* 1:570–578.

Lombard J, López-García P, Moreira D. 2012. Phylogenomic investigation of phospholipid synthesis in archaea. *Archaea* 2012:630910. DOI: 10.1155/2012/630910.

Ohnuma S, Hirooka K, Hemmi H, Ishida C, Ohto C, Nishino T. 1996. Conversion of product specificity of archaebacterial geranylgeranyl-diphosphate synthase. *Journal of Biological Chemistry.* 271:18831–18837.

Ohnuma S, Hirooka K, Tsuruoka N, Yano M, Ohto C, Nakane H, Nishino T. 1998. A pathway where polyprenyl diphosphate elongates in prenyltransferase. *Journal of Biological Chemistry.* 273:26705–26713.

Tachibana A, Yano Y, Otani S, Nomura N, Sako Y, Taniguchi M. 2000. Novel prenyltransferase gene encoding farnesylgeranyl diphosphate synthase from a hyperthermophilic archaeon, *Aeropyrum pernix*. *European Journal of Biochemistry.* 267:321–328.

Suppl. Figure 3

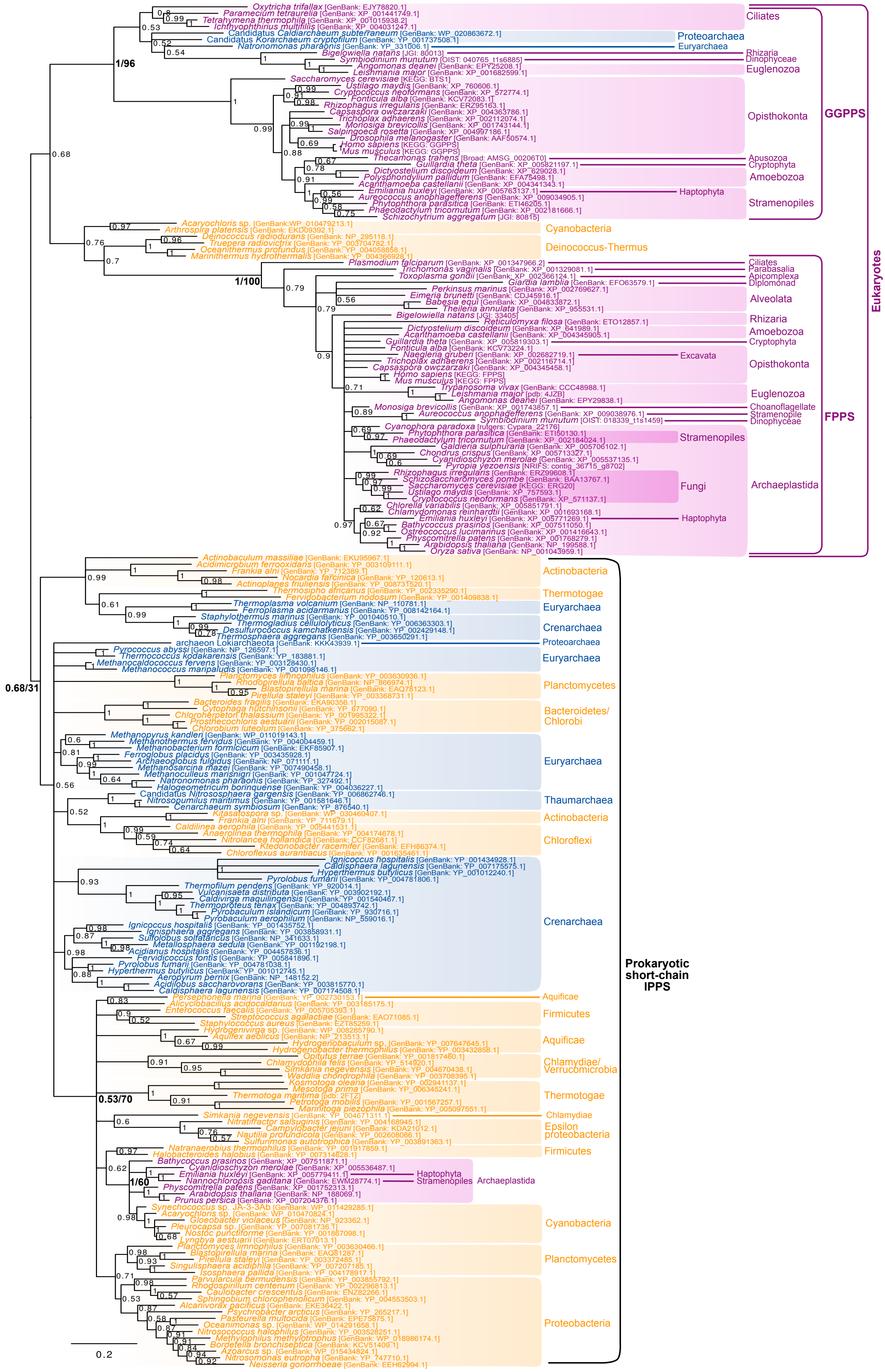

Supplement: Figure S3 [file peerj-04-2626-s003.pdf]
