# Supplementary material for: Early evolution of polyisoprenol biosynthesis and the origin of cell walls
Source: PeerJ. 2016 Oct 26;4:e2626. doi: 10.7717/peerj.2626 (PMC5088576; doi:10.7717/peerj.2626)

Suppl. Figure 4. Bayesian phylogeny of the undecaprenyl pyrophosphate phosphatase (UppP). Tree is unrooted and reconstructed using 171 sequences and 190 conserved sites. Multifurcations correspond to branches with Bayesian posterior probabilities <0.5, whereas numbers at nodes indicate Bayesian posterior probabilities higher than 0.5. Colors on leaves represent the affiliation of sequences to their respective domain of life: archaea (blue) and bacteria (orange).

It was impossible to detect any significant UppP homologues in eukaryotes even when an HMM profile was built from a prokaryotic alignment to look for distant homologues (see Methods). On the contrary, some archaeal and a wide diversity of bacterial homologues were identified. The phylogeny of the UppP homologues shows that most archaeal sequences cluster within a monophyletic group (BPP = 0.96), and that others are likely xenologues derived from bacteria. The crenarchaea form a monophyletic group within the archaeal clade, but the other proteoarchaea to which they are frequently associated are scattered among the paraphyletic euryarchaeal sequences. Such discrepancies with regard to the taxonomic groups can be explained by inter-archaeal HGT and/or reconstruction errors due to a lack of phylogenetic signal. Yet, the fact that a wide diversity of archaea have UppP homologues that cluster in a monophyletic group suggests the presence of this gene in LACA. This enzymes are good candidates for *de novo* polyprenol pyrophosphatase activity in archaea, a hypothesis that would be worth testing experimentally. With regard to bacterial UppP homologues, it is noteworthy that, in spite of many obvious xenologues and the poor resolution in some basal nodes, a lot of sequences cluster according to the main bacterial taxa. This supports the ancestral origin of UppPs in bacteria. Importantly, the presence of UppP homologues in the respective ancestors of both archaea and bacteria may suggest a cenancestral origin of UppP, thus supporting the idea that the cenancestor could have synthesized functional bacterial-like polyisoprenol lipid carriers.

Suppl. Figure 4.

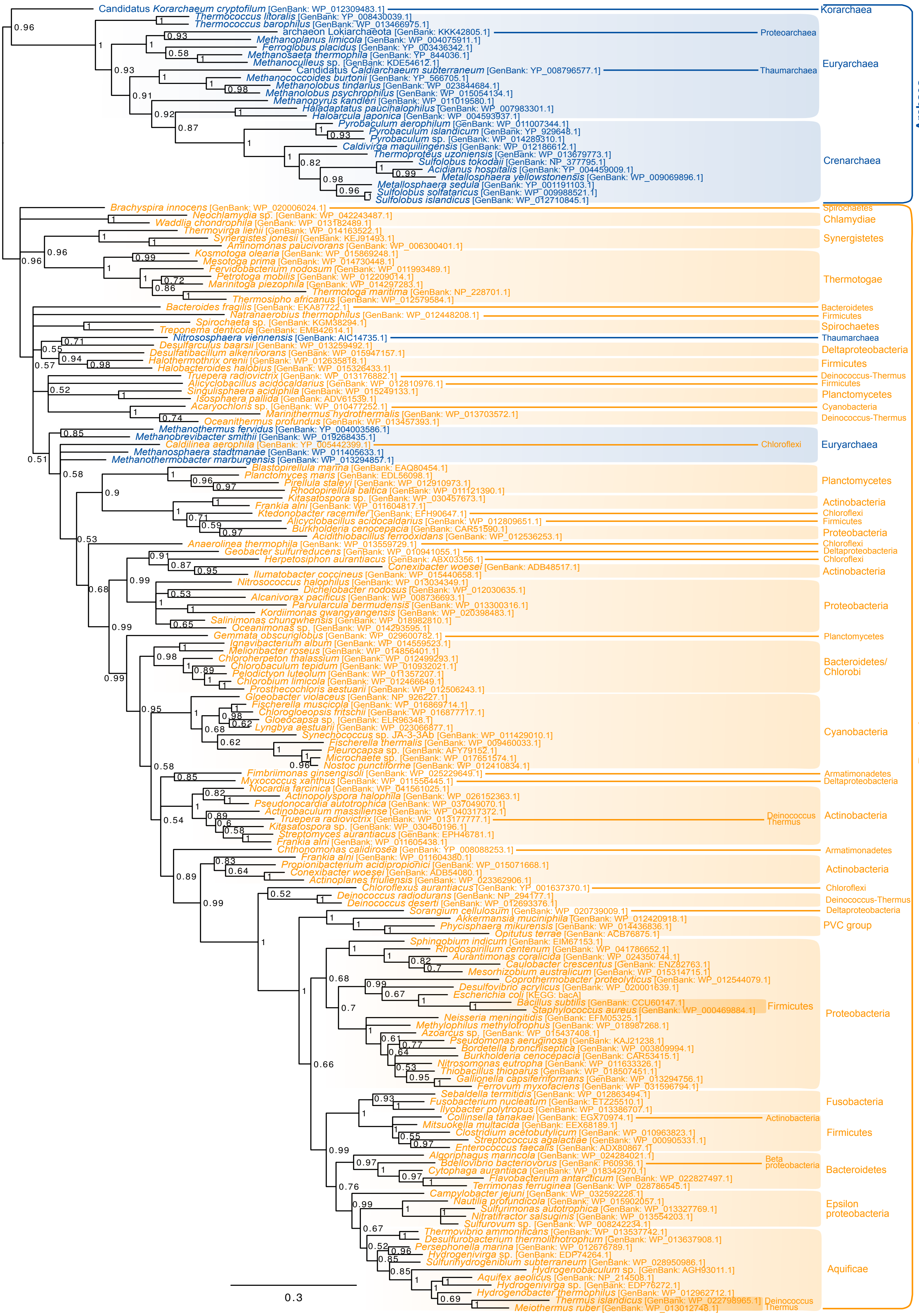

Supplement: Figure S4 [file peerj-04-2626-s004.pdf]
