# Supplementary material for: Early evolution of polyisoprenol biosynthesis and the origin of cell walls
Source: PeerJ. 2016 Oct 26;4:e2626. doi: 10.7717/peerj.2626 (PMC5088576; doi:10.7717/peerj.2626)

Suppl. Figure 5. Bayesian phylogeny of the PAP-motif phosphatases. Tree is unrooted and reconstructed using 175 sequences and 124 conserved sites. Multifurcations correspond to branches with Bayesian posterior probabilities <0.5, whereas numbers at nodes indicate Bayesian posterior probabilities higher than 0.5. Colors on leaves represent the affiliation of sequences to their respective domain of life: archaea (blue), bacteria (orange) and eukaryotes (purple).

Homologues of the PAP-motif phosphatase family are widespread among the three domains of life. The eukaryotic sequences are monophyletic (BPP = 1) with a duplication in the chlorophytes, but the relationships among the prokaryotic sequences are unclear. The tree is poorly resolved and, despite some groups that correspond to particular prokaryotic taxa, it is dominated by many paralogues and xenologues. This is unsurprising because these genes are already known to be frequently duplicated and to have poor enzymatic specificity that could encourage neofunctionalization and transfer events (El Ghachi et al., 2005; Touzé, Blanot & Mengin-Lecreulx, 2008; Bickford & Nick, 2013). This result does not allow determining if the origin of this function was cenancestral or more recent. Despite the lack of resolution in the eukaryotic clade, its monophyly suggests the presence of this gene in LECA. The former origin of the eukaryotic PAP-motif phosphatases is elusive.

Bickford JS, Nick HS. 2013. Conservation of the PTEN catalytic motif in the bacterial undecaprenyl pyrophosphate phosphatase, BacA/UppP. *Microbiology.* 159:2444–55. DOI: 10.1099/mic.0.070474-0.

El Ghachi M, Derbise A, Bouhss A, Mengin-Lecreulx D. 2005. Identification of multiple genes encoding membrane proteins with undecaprenyl pyrophosphate phosphatase (UppP) activity in *Escherichia coli*. *Journal of Biological Chemistry.* 280:18689–95. DOI: 10.1074/jbc.M412277200.

Touzé T, Blanot D, Mengin-Lecreulx D. 2008. Substrate specificity and membrane topology of *Escherichia coli* PgpB, an undecaprenyl pyrophosphate phosphatase. *Journal of Biological Chemistry.* 283:16573–83. DOI: 10.1074/jbc.M800394200.

Suppl. Figure 5.

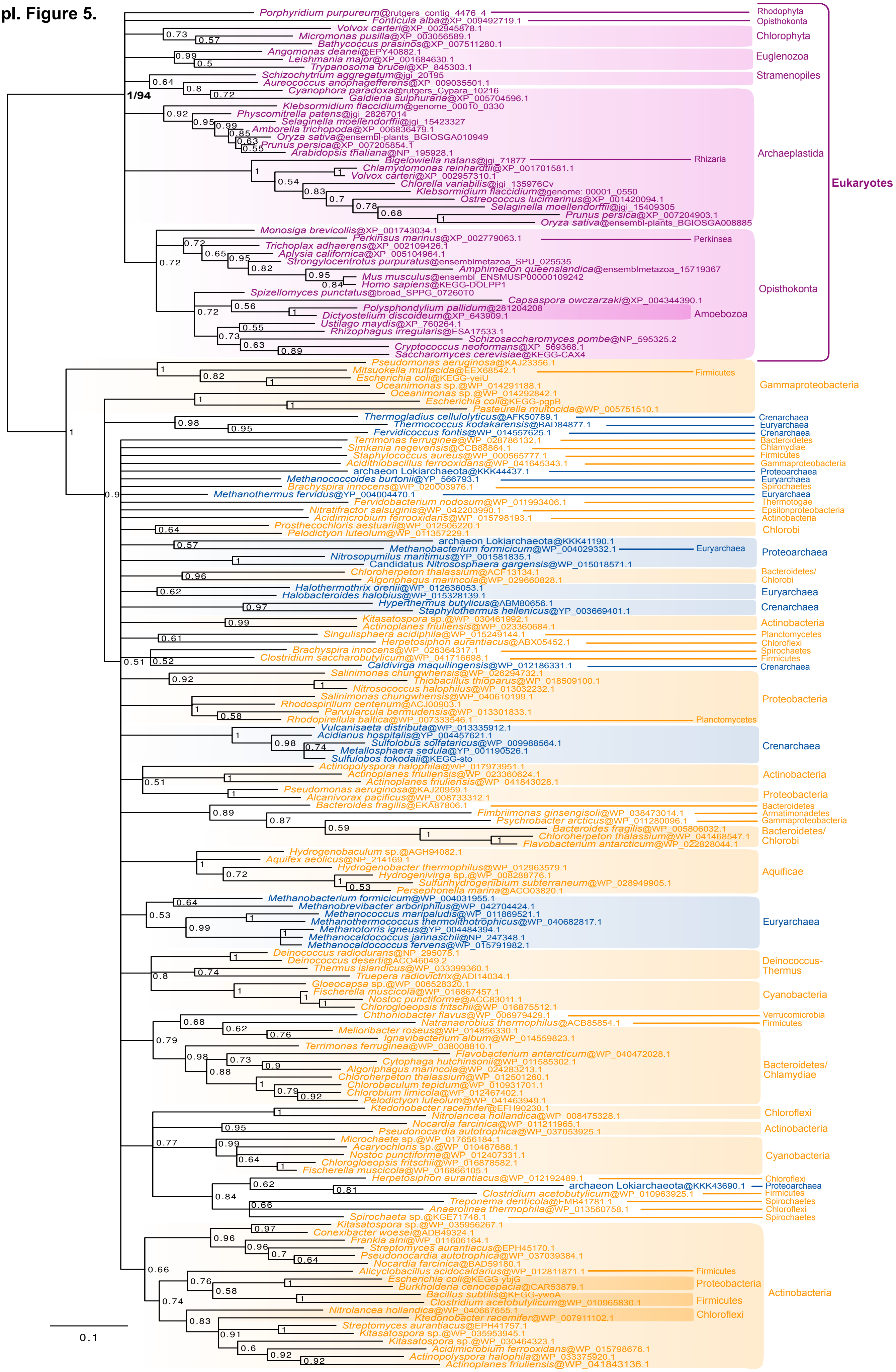

Supplement: Figure S5 [file peerj-04-2626-s005.pdf]
