# Supplementary material for: Early evolution of polyisoprenol biosynthesis and the origin of cell walls
Source: PeerJ. 2016 Oct 26;4:e2626. doi: 10.7717/peerj.2626 (PMC5088576; doi:10.7717/peerj.2626)

Suppl. Figure 6. Polyprenol reductases. A. Schematic phylogenetic tree of the 5α-reductases and ICMTs. FastTree phylogeny was reconstructed using 308 sequences and 112 conserved sites. Branch colors represent the affiliation of sequences to their respective domain of life: archaea (blue), bacteria (orange) and eukaryotes (purple). **B. Bayesian phylogeny of the 5α-reductases.** Tree is unrooted and reconstructed using 130 sequences and 132 conserved sites. Multifurcations correspond to branches with Bayesian posterior probabilities <0.5, whereas numbers at nodes indicate Bayesian posterior probabilities higher than 0.5. Colors on leaves represent the affiliation of sequences to their respective domain of life: archaea (blue), bacteria (orange) and eukaryotes (purple).

The enzyme responsible for the α-unit saturation has not been described in archaea yet, but it has been characterized in several opisthokonts (human, mouse and yeast, (Cantagrel et al., 2010)). The human polyprenol reductase is called Srd5A3 and it belongs to a large family of steroid 5α-reductases, some of which (Srd5A1 and Srd5A2) are well characterized and known to be involved in testosterone reduction (Russell & Wilson, 1994). A previous analysis (restricted to opisthokonts and *Arabidopsis thaliana*) concluded that the ancestral substrate of the three paralogues could have been something other than a steroid (Cantagrel et al., 2010), but the actual origin of these genes in eukaryotes was not assessed.

A first round of BLASTp searches did not find archaeal homologues of these genes, but only a large diversity of eukaryotic and a few bacterial sequences. These sequences were used to build an HMM profile to look for distant homologues in archaea and bacteria (see Methods). In this search, some distant homologues were detected in both prokaryotic domains, and included some isoprenylcysteine carboxyl methyltransferases (ICMTs), which are responsible for the carboxyl methylation of proteins (Romano & Michaelis, 2001). As a result, a preliminary analysis was simultaneously carried out on 5α-reductases and ICMTs from the three domains of life (Part A of the figure). This tree shows a clear phylogenetic split between both functions (BPP = 1). The phylogeny of the ICMTs is a matter for another analysis, but this result confirmed that the distant prokaryotic homologues detected are more closely related to ICMT and, hence, they are more likely to carry out that function. In other words, no archaeal, and only a few bacterial sequences can be assumed to have a close relationship to the eukaryotic 5α-reductases. This implies that archaea use an alternative mechanism to saturate the α-unit of their Dol-P.

The specific 5α-reductase phylogeny found the previously observed split (BPP = 1) between polyprenol reductase homologues (Srd5A3) and other steroid 5α-reductases (Srd5A1/2, (Cantagrel et

al., 2010)). Both paralogues are widespread among eukaryotes. Even though not all relationships are well resolved, the phylogeny suggests that the duplication of these genes predated LECA, with subsequent duplications happening later in some eukaryotic lineages. This further implies that the polyprenol reductases were specifically evolved in the eukaryotic lineage–not from prokaryotes–to carry out their polyprenol saturation. All the bacterial sequences branched among the other steroid 5α-reductases, thus suggesting that these bacteria acquired their genes from eukaryotes in the recent past. This is not totally surprising, as some bacterial organisms are known to desaturate the eukaryotic hormones of their hosts (Levy & Talalay, 1959), but it is intriguing because the dehydrogenases described so far were not homologous to the 5α-reductase family (Florin et al., 1996).

Cantagrel V, Lefeber DJ, Ng BG, Guan Z, Silhavy JL, Bielas SL, Lehle L, Hombauer H, Adamowicz M, Swiezewska E, De Brouwer AP, Blümel P, Sykut-Cegielska J, Houliston S, Swistun D, Ali BR, Dobyns WB, Babovic-Vuksanovic D, van Bokhoven H, Wevers R a, Raetz CRH, Freeze HH, Morava E, Al-Gazali L, Gleeson JG. 2010. SRD5A3 is required for converting polyprenol to dolichol and is mutated in a congenital glycosylation disorder. *Cell.* 142:203–17. DOI: 10.1016/j.cell.2010.06.001.

Florin C, Köhler T, Grandguillot M, Plesiat P. 1996. Comamonas testosteroni 3-Ketosteroid-delta(5alpha)-dehydrogenase: gene and protein characterization. *Journal of Bacteriology.* 178:3322–3330.

Levy HR, Talalay P. 1959. Bacterial oxidation of steroids : II . Studies on the enzymatic mechanism of ring A dehydrogenation. *Journal of Biological Chemistry.* 234:2014–2021.

Romano JD, Michaelis S. 2001. Topological and mutational analysis of *Saccharomyces cerevisiae* Ste14p, founding member of the isoprenylcysteine carboxyl methyltransferase family. *Molecular Biology and Evolution.* 12:1957–1971.

Russell DW, Wilson JD. 1994. Steroid 5-alpha-reductase: two genes/two enzymes. *Annual Review Biochemistry.* 63:25–61.

Suppl. Figure 6

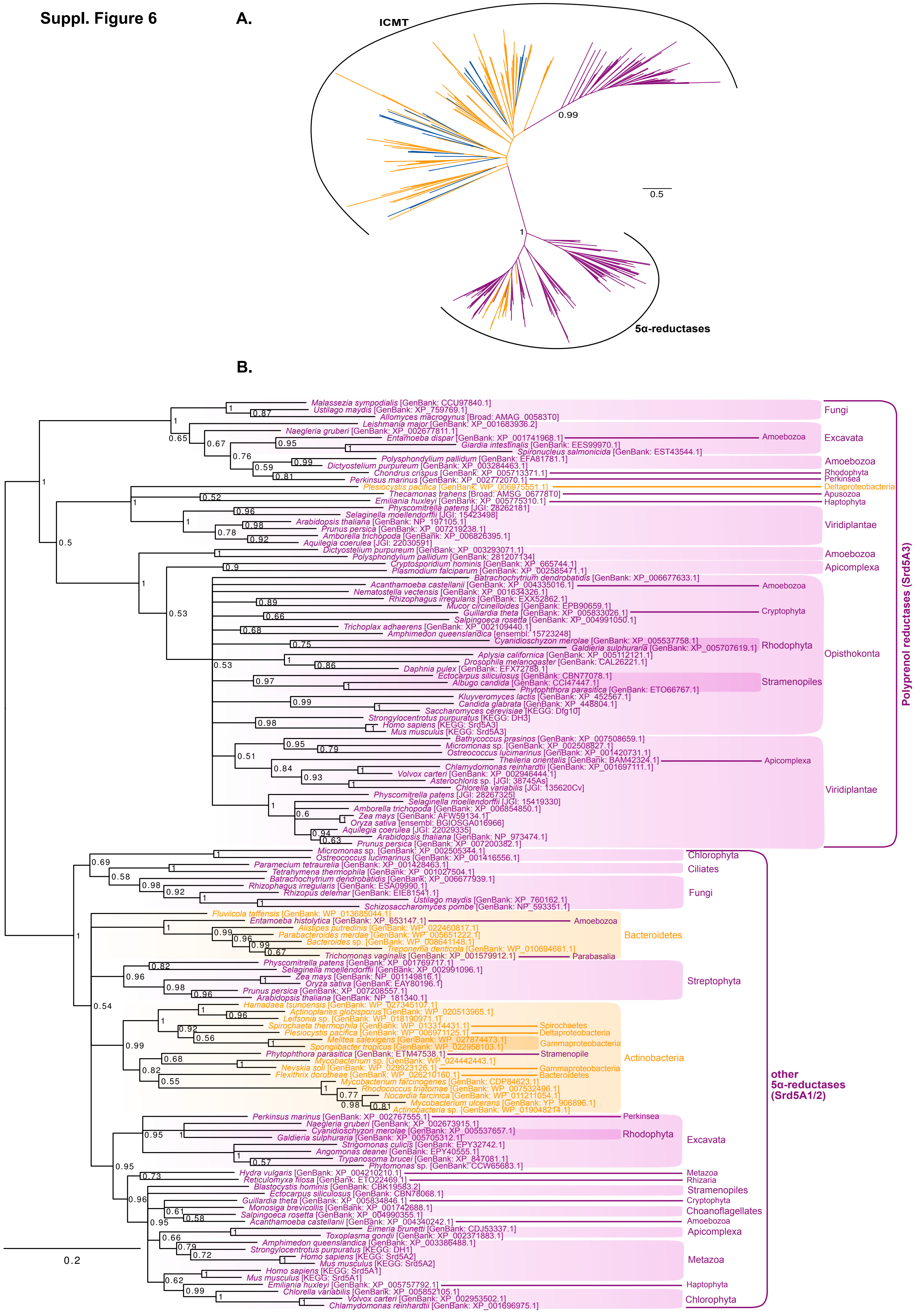

Supplement: Figure S6 [file peerj-04-2626-s006.pdf]
