# Supplementary material for: Early evolution of polyisoprenol biosynthesis and the origin of cell walls
Source: PeerJ. 2016 Oct 26;4:e2626. doi: 10.7717/peerj.2626 (PMC5088576; doi:10.7717/peerj.2626)

Suppl. Figure 7. FastTree phylogeny of the dolichol kinases. Tree is unrooted and reconstructed using 236 sequences and 83 conserved sites. Multifurcations correspond to branches with support values <0.5, whereas numbers at nodes indicate support values higher than 0.5. Colors on leaves represent the affiliation of sequences to their respective domain of life: archaea (blue), bacteria (orange) and eukaryotes (purple).

Homologues of the dolichol kinase were easily detected among a large diversity of eukaryotes, but the basic BLASTp only detected a few prokaryotic sequences. A psi-BLAST search was respectively applied to the archaeal and bacterial genomes in order to look for more distant homologues. The psi-BLAST searches were reiterated for a few cycles until no more new sequences were found. A protein domain analysis (data not shown) revealed that most bacterial and a few archaeal sequences contained a CTP transferase domain characteristic of the CDP-diacylglycerol synthases (CdsA) involved in phospholipid synthesis (Sparrow & Raetz, 1985). This observation was expected, as dolichol kinases are known to contain a motif characteristic of CTP-dependent cytidylyltransferases, i.e. enzymes that use CTP to attach a CDP to a large diversity of molecules (Kanehara et al., 2015). The CdsA homologues were included in the analyses of the dolichol kinases because they are their closest relatives and, contrary to dolichol kinases, CdsA genes are known to be widespread in the three domains of life and were probably already present in the cenancestor (Lombard, López-García & Moreira, 2012).

The eukaryotic dolichol kinases, their closest relatives and the putative archaeal CdsA genes are very divergent. The alignment is poor and unreliable. The resulting phylogenies change widely depending on the sequences or alignment positions selected for the phylogenetic reconstruction (data not shown). Thus, these phylogenies should be considered with caution. Four groups are consistently recovered among all tentative phylogenies: three groups of monophyletic CdsA genes–one for each domain of life (BS > 0.84)–, and one group of eukaryotic dolichol kinases (BS = 0.87). A supplementary group of mixed archaeal, bacterial and eukaryotic sequences was found to be basal to the dolichol kinases (in other trees, these sequences formed a sister group with the dolichol kinases). The main hint that we may have about the origin of the eukaryotic dolichol kinases is that most of the non-CdsA prokaryotic sequences that are basal to eukaryotes are proteoarchaeal, which may support a closest relationship between proteoarchaea and eukaryotes. Yet, the phylogenetic signal is so poor that the position of these prokaryotic sequences could simply reflect a phylogenetic reconstruction artifact due to the high divergence of these sequences with regard to the CdsA genes. Nothing is known about the proteoarchaeal sequences in this tree, so the characterization of these proteins will be very helpful

to evaluate the plausibility of their relationship to the eukaryotic dolichol kinases. Finally, another intriguing question is how euryarchaea phosphorylate their dolichol molecules, as most euryarchaeal genomes seem to lack the closest homologues of the dolichol kinases.

Kanehara K, Cho Y, Lin Y-C, Chen C-E, Yu C-Y, Nakamura Y. 2015. *Arabidopsis* DOK1 encodes a functional dolichol kinase involved in reproduction. *Plant J.* 81:292–303. DOI: 10.1111/tpj.12727.

Lombard J, López-García P, Moreira D. 2012. Phylogenomic investigation of phospholipid synthesis in archaea. *Archaea* 2012:630910. DOI: 10.1155/2012/630910.

Sparrow CP, Raetz CR. 1985. Purification and properties of the membrane-bound CDP-diglyceride synthetase from *Escherichia coli*. *Journal of Biological Chemistry.* 260:12084–91.

Suppl. Figure 7

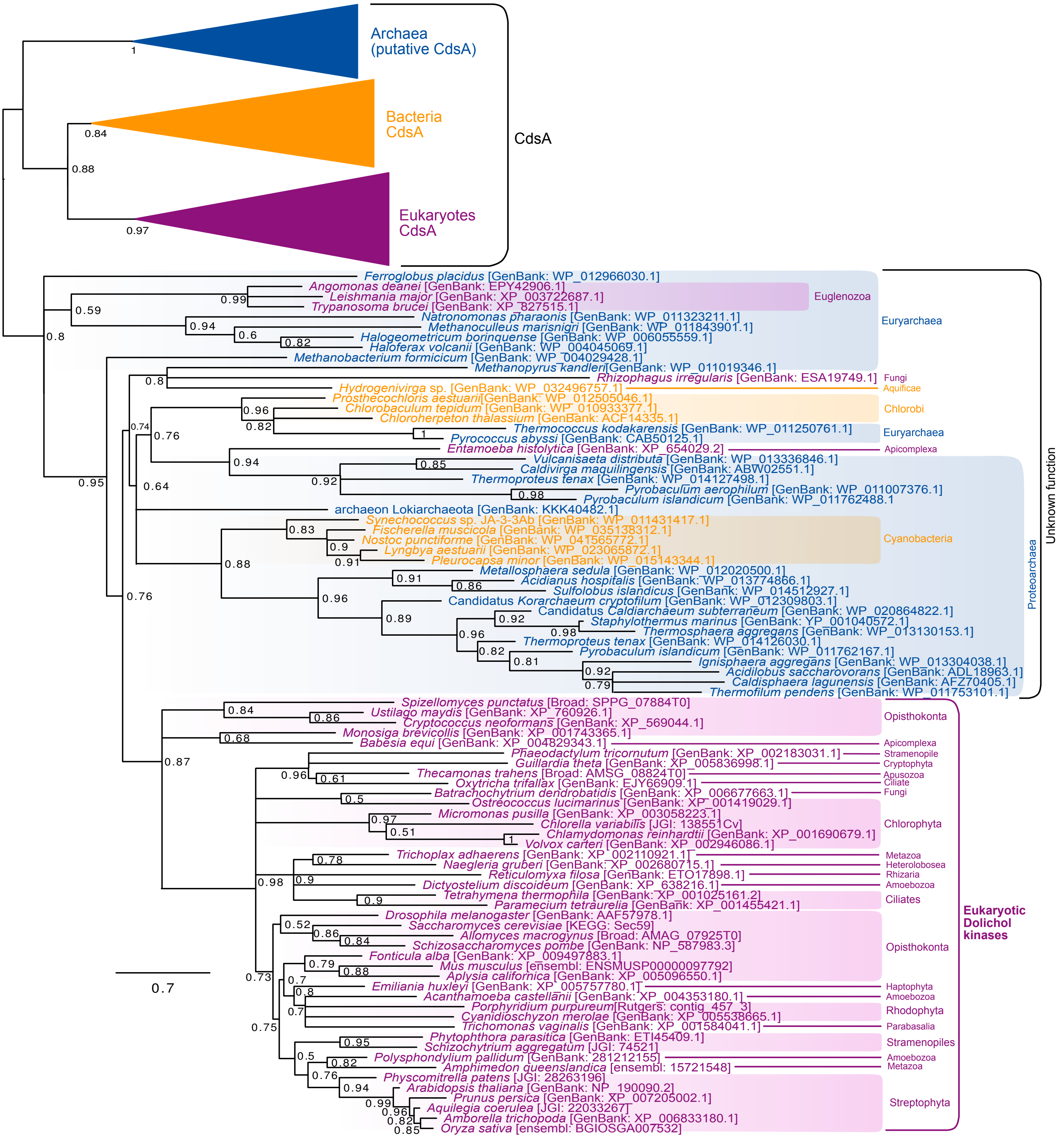

Supplement: Figure S7 [file peerj-04-2626-s007.pdf]
